# Evaluation method for ecology-agriculture-urban spaces based on deep learning

**DOI:** 10.1038/s41598-024-61919-1

**Published:** 2024-05-18

**Authors:** Anqi Li, Zhenkai Zhang, Zenglin Hong, Lingyi Liu, Yuanmin Liu

**Affiliations:** 1 School of Land Engineering, Chang’an University, Xi’an, 710054 China; 2 Shaanxi Satellite Application Center for Natural Resources, Shaanxi Institute of Geological Survey, Xi’an, China; 3 Satellite Remote Scensing Application Centre, CESS, China-SCO, Xi’an, 710054 China; 4 Shaanxi Urban Geology and Underground Space Engineering Technology Research Center, Xi’an, 710054 China; 5 School of Computer Science, National Engineering Laboratory for Integrated Aero-Space-Ground-Ocean Big Data Application Technology, Shaanxi Provincial Key Laboratory of Speech Image Information Processing, Northwestern Polytechnical University, Xi’an, 710129 China; 6 The 41St Institute of the Fourth Academy of CASC, Xi’an, 710025 China

**Keywords:** Territorial space, Suitability evaluation, Deep learning, SARes-NET, Ecology, Environmental sciences, Environmental social sciences

## Abstract

With the increasing global population and escalating ecological and farmland degradation, challenges to the environment and livelihoods have become prominent. Coordinating urban development, food security, and ecological conservation is crucial for fostering sustainable development. This study focuses on assessing the "Ecology-Agriculture-Urban" (E-A-U) space in Yulin City, China, as a representative case. Following the framework proposed by Chinese named "environmental capacity and national space development suitability evaluation" (hereinafter referred to as "Double Evaluation"), we developed a Self-Attention Residual Neural Network (SARes-NET) model to assess the E-U-A space. Spatially, the northwest region is dominated by agriculture, while the southeast is characterized by urban and ecological areas, aligning with regional development patterns. Comparative validations with five other models, including Logistic Regression (LR), Naive Bayes (NB), Gradient Boosting Decision Trees (GBDT), Random Forest (RF) and Artificial Neural Network (ANN), reveal that the SARes-NET model exhibits superior simulation performance, highlighting it’s ability to capture intricate non-linear relationships and reduce human errors in data processing. This study establishes deep learning-guided E-A-U spatial evaluation as an innovative approach for national spatial planning, holding broader implications for national-level territorial assessments.

## Introduction

Territorial space development suitability evaluation refers to the degree of suitability of national space for different development and utilization purposes such as ecological protection, agricultural production, and urban development and construction. It’s basic concept originated from land suitability assessment. In 1976, the Food and Agriculture Organization of the United Nations (FAO) formulated the “Outline of Soil Evaluation”, proposing the classification of land based on suitability, serving the purpose of land use planning^1^. Subsequently, countries around the world, building upon this outline, proposed research frameworks tailored to optimize regional functions based on their respective contexts^2^. In addition to intrinsic studies within geography, encompassing research on natural geography divisions, human geography divisions, and comprehensive geographic divisions, there are two main aspects to consider. One category is rooted in land science, involving research on land (use) systems, such as spatial configuration of agricultural production systems^3^, identification of urban expansion and reasonable development boundaries^4,5^, and analysis of conflicts and rationality of farmland systems and urban expansion^6^. Essentially, these studies aim to achieve optimal land allocation schemes that satisfy overall suitability for cultivation or construction^7,8^. The other category is grounded in ecology, focusing on ecosystem service zoning, such as delineation of natural reserves and zoning of ecological functions^9,10^. Clearly, to achieve an integrated optimization solution in regional functional zoning that ensures optimal urbanization, food, and ecological security, these two types of studies need to be integrated first^11^. The "Dual Evaluation" policy proposed by the Chinese government is an attempt of this approach. Some scholars use the "National space development suitability evaluation" within the "Dual Evaluation" framework to provide operational evaluation strategies for scientifically arranging ecological space, agricultural space, and urban space^4,12–15^.

Research on the national space development suitability evaluation primarily involves the construction of evaluation indicator systems, evaluation methods, and calculation models. The selection of evaluation indicators is crucial, and based on existing research, the assessment of ecological importance is derived from the overlay of results from the importance assessment of ecosystem services and ecological sensitivity^3,16,17^. Agricultural evaluation typically relies on the region's soil and water resources, integrating climate and environmental assessment results to determine preliminary results for agricultural production suitability levels^17,18^. Urban evaluation similarly uses soil and water resources as a foundation, integrating climate, environmental, and locational assessment results. Correction of suitability level results for urban construction is performed using indicators of disaster risk and locational advantage^19,20^. With the increasing application of big data, many scholars, in expanding the indicator system, have focused on integrating POI data indicators into suitability evaluations, enriching the social-level indicator system and making it more regionally specific^21^. The establishment of methods for evaluating indicators primarily employs approaches such as expert experience method^16^, extreme value method^22,23^, and principal component analysis^24^. Regarding the assignment of weights, methods include expert scoring^6,25–27^, Analytic Hierarchy Process (AHP)^28^, entropy weight method^26,29^, or adopting the "weakest link" principle, implementing a "single veto" for the weakest indicator. While these methods are practical and allow for quantifiable results, they are subject to a significant degree of subjectivity. For example, the accuracy of the entropy weight method is greatly influenced by data quality^30^. The AHP is susceptible to cognitive biases from expert scholars^31^. Additionally, in situations with a multitude of evaluation indicators, increased steps may lead to logical errors.

In recent years, the application of artificial intelligence (AI) in national space development suitability evaluation has gained prominence due to its automated decision-making capabilities. AI, with its ability to comprehend complex nonlinear relationships and handle dynamic system data more effectively, has demonstrated superior performance and higher accuracy compared to traditional methods in various studies. Notably, machine learning techniques such as Logistic Regression(LR)^32^, Naive Bayes (NB)^22^, Gradient Boosting Decision Trees (GBDT)^33^, and Random Forests (RF)^21^ have been widely utilized in national space development suitability. For instance, Xia et al.^34^ and Huang et al.^35^ employed the ‘quadtree’ algorithm and ‘XGBoost’ algorithm, respectively, to assess suitability in different regions, achieving favorable classification results aligning with regional development needs. Zhao et al.^22^ explored the application of NB machine learning in urban suitability assessment at the county level. In comparison to the linear expert-based evaluation following the "Dual Evaluation", the rationality of the NB network evaluation substantially surpassed that of the linear expert-based evaluation, indicating a higher feasibility of the evaluation results.

Compared to machine learning, deep learning models exhibit robust nonlinear modeling capabilities, providing enhanced adaptability to intricate geographical phenomena and planning issues. Research in this domain highlights the adeptness of deep learning in capturing intricate nonlinear relationships between evaluation factors and suitability levels, thereby mitigating human errors in data processing^5,36,37^. Wei et al.^38^ assessed the applicability of multiple AI models for predicting suitable areas for agricultural zones in Yunnan Province. The study compared the performance of Support Vector Machines (SVM), k-Nearest Neighbor (kNN), Back Propagation Neural Network (BPNN), Convolutional Neural Networks (CNN), and the The Feature Attention (FA) + Residual Neural Network (ResNet) model in the multi-class prediction of agricultural suitability zones in Yunnan Province. The FA + ResNet model, incorporating a residual network with attention mechanisms, demonstrated superior performance in predicting suitable areas for agricultural suitability zones. The residual structure effectively integrates features from different layers, significantly improving the classification performance of the model. Given that territorial space is a complex system where natural and socio-economic factors interact, involving high-dimensional and intricate spatial information, it’s evaluation is a multivariate nonlinear problem. The self-attention structure can adaptively weight features from each hidden layer, filtering out irrelevant ones. Based on these considerations, we developed the Self-Attention Residual Neural Network (SARes-NET) model to evaluate territorial space development suitability.

The objectives of this study were to determine: 1. In the evaluation of E-A-U space, we borrowed and extended the "National Space Development Suitability Evaluation" system, constructing a corresponding dataset. 2. We autonomously developed the SARes-NET model to assess E-A-U space, using GIS technique for spatial representation of the evaluation results. 3. To demonstrate the suitability of the SARes-NET method for this task, the research results were compared and validated against five machine learning algorithms (LR, NB, GBDT, RF) and deep learning algorithms (ANN). This study aims to explore a new evaluation method suitable for E-A-U space, enhance evaluation accuracy, and provide reference for similar research endeavors.

## Materials and methods

### Study area

**Figure 1:**
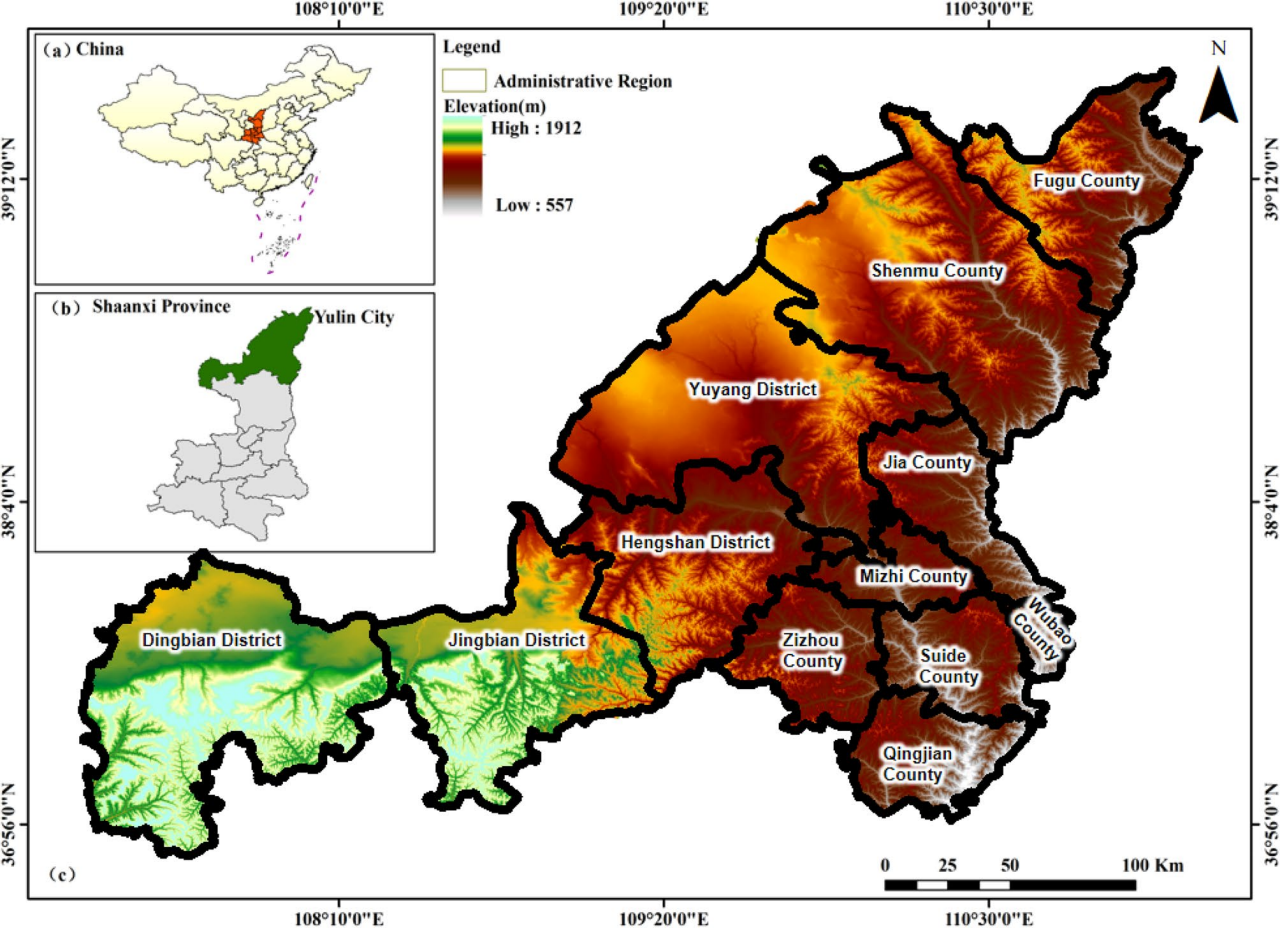
Study area is the City of Yulin, Shaanxi, China: (**a**) Administrative map of China, (**b**) Administrative map of Shaanxi Province, (**c**) Digital elevation map of Yulin City. Note that we use QGIS sofware (https://qgis.org/en/site/, version: 3.34) for plotting.

Yulin City (Fig. 1), situated in the northernmost region of Shaanxi Province, China, is an inland city characterized by an expansive land area of 42,920.2 square kilometers. The terrain within this city is notable for its elevation variations, stretching from highlands in the northwest to lower areas in the southeast. Yulin City experiences a temperate arid and semi-arid continental monsoon climate, featuring four distinct seasons. This region comprises a northern sandy grass beach area and a southern loess hilly and gully region.In recent years, Yulin City has witnessed remarkable improvements in its ecological environment, concomitant with rapid economic growth. However, this progress has come with the challenge of balancing economic and social development with ecological protection, given the city's location at the interface between the Loess Plateau and the Mu Us Sandy Land. It falls within the category of a typical agro-pastoral ecotone, characterized by heightened ecological sensitivity and a relatively fragile environmental ecosystem. Addressing the long-term challenge of harmonizing economic and social development with the preservation of the ecological environment and guiding the layout of urban spaces will be a central concern for Yulin City. Serving as a vital energy and chemical hub in China, a key ecological shield in the midstream region of the Yellow River Basin, and a model area for ecological civilization within the Loess Plateau, this region holds a unique and multifaceted role in the broader context.

### Data sources and processing

This study utilizes diverse data sources, encompassing basic geographic, land and natural resources, location conditions, socio-economic, and climate data, all sourced from 2020. Employing QGIS 3.34, the coordinate system is standardized to CGCS2000 projection, with a spatial scale of 90 m × 90 m, resulting in 5,298,221 evaluation units. For detailed information on data sources and processing, refer to Table 1. The land use data were reclassified into six categories: arable land, forest land, grassland, water area, construction land, and unused land using QGIS 3.34. Precipitation data were spatially distributed using Kriging interpolation to create annual precipitation distribution data. Location condition data were generated using Euclidean distance analysis to represent the distance distribution of different road types. Population data were corrected based on the 2020 statistical yearbook of Yulin City, following the method outlined by Liu and Hu^39^. Point of Interest (POI) data were transformed into spatial data using kernel density analysis.

**Table 1:** Data Type and Sources comprising basic geographic, land resource, natural resource, location conditions, socio-economic, and meteorological data.

Data type	Name		Source
Basic geographic data	Administrative boundary		Provinces, cities and counties data download network (https://www.shengshixian.com/)
	DEM		Geospatial data cloud (http://www.gscloud.cn)
	Slope		Calculated by DEM
	Slope aspect		
Land resource data	Soil quality		Resource and Environment Science and Data Center(http://www.resdc.cn/)
	Soil erosion		
	Land use types		
	Ecological Protection Red Line		From official data
	Nature reserves		
	Drinking water source		
	Permanent basic farmland		
	Urban built-up area		
Natural resource data	Distribution of high-risk Geological hazards area		
	NPP		Resource and Environment Science and Data Center (http://www.resdc.cn/)
	NDVI		
	Average precipitation		China 's surface climate data daily data set V3.0
Location conditions data	Distance to roads		Open Street Map
	Distance to river		
Socio-economic data	Population density		WorldPoP database(https://www.worldpop.org/)
	Night lighting		^40^
	POI	Distribution of healthcare regions	Tencent maps
		Distribution of Scenic spots	
		Distribution of educational institutions	
		Distribution of catering establishment	
Meteorological data	Average annual accumulated temperature		Resource and Environment Science and Data Center(http://www.resdc.cn/)
	wetness index		

### Feature engineering

Following the principles of scientific rigor, representation, and accessibility in feature selection, we delineate essential feature factors in Table 2 (Supplementary Fig. S1 online). Drawing insights from prior research and accounting for the unique ecological dynamics of the Yulin region, we judiciously curated a comprehensive set of 22 factors. These include pivotal indicators such as soil erosion, NDVI, and NPP. For ecological space, we meticulously selected land resources and natural resources, constituting assessment features^3,16,25^. In the domain of agricultural space, our focus extended to land resources, water resources, climate conditions, and environmental variables—comprising agricultural space evaluation features^17,27^. Delving into urban space, bound by defined development parameters, we incorporated fundamental geographical data (terrain and slope) and integrated socio-economic metrics gleaned from Point of Interest (POI) datasets, alongside climate data form the assessment features^19,20^.

**Table 2:** Ecological, Agricultural, Urban space evaluation index and feature engineering selection.

Spatial classification	Type of evaluation index	Feature engineering	
Ecological space	Land resource data	Soil erosion (sand, clay, silt)	
	Natural resource data	NDVI	
		NPP	
	Location conditions	Distance to river	
Agricultural space	Basic geographic data	DEM	
		Slope	
		Slope aspect	
	Natural resource data	Average precipitation	
	Land resource data	Land use types	
		Soil quality	
	Meteorological data	Average annual accumulated temperature	
Urban space	Natural resource data	Distribution of high-risk Geological hazards area	
	Location conditions	Distance to roads	
	Meteorological data	wetness index	
	Socio-economic data	POI	Distribution of healthcare regions
			Distribution of Scenic spots
			Distribution of educational institutions
			Distribution of catering establishment
		Night lighting	
		Population density	

### Model interpretation

1. *Logistic regression* A linear model employed for solving classification problems. It models the probability of an event occurrence by taking the logarithm of the odds (log-odds), which is a linear combination of one or more independent variables. It performs well in exploring linear relationships^32^.
2. *Naive Bayes* A simple and effective classification algorithm based on Bayes' theorem. It leverages the assumption of independence between features to assign an instance to the category with the highest probability. It is suitable for large-scale datasets and high-dimensional feature spaces, exhibiting relative simplicity and good adaptability to high-dimensional data^22^.
3. *Gradient Boosting Decision Trees (GBDT)* GBDT is an ensemble learning method that enhances model performance through iterative training of decision trees using gradient boosting. It is applicable to regression and classification problems, capturing nonlinear relationships in data. With strong fitting capabilities, it performs well on complex datasets^33^.
4. *Random Forest (RF)* An ensemble learning method that mitigates overfitting by training multiple decision trees and aggregating their voting results. Used for both classification and regression, RF demonstrates good performance on high-dimensional and large-scale datasets^21,41^.
5. *Artificial Neural Network (Ann)* A model designed based on neural network architecture, capable of complex nonlinear modeling through multiple layers of neurons. Suitable for tasks such as handling large-scale data, image processing, and natural language processing, it exhibits powerful fitting capabilities^5,42,43^.
6. Self-Attention Residual Neural Network Diagram (SARes-NET)

**Figure 2:**
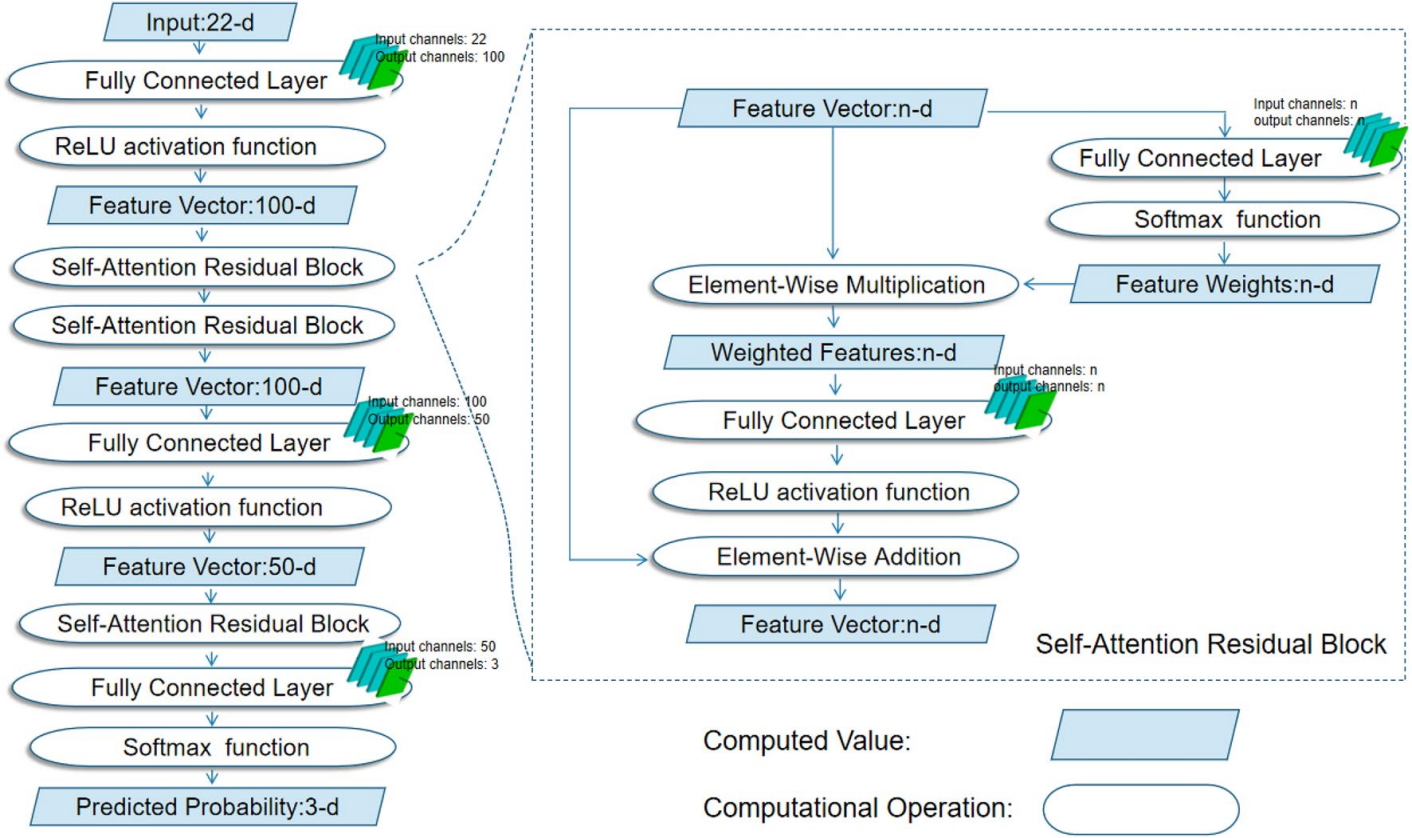
Self-Attention Residual Neural Network Diagram (SARes-NET), Utilizing 22 features, forming a feature vector. The network elevates feature dimension to 100, then undergoes two self-attention residual modules for nonlinear computations, reducing dimension to 50. This densified feature vector is processed by fully connected layers to calculate scores for three land-use categories, transformed into predicted probabilities via softmax function.

We designed a deep neural network model structure based on the characteristics of the data, as depicted in Fig. 2. We will select 22 features, including basic geographic data such as terrain, slope, and aspect, land resource data like soil erosion, soil texture, and land use, natural resource data like NPP, NDVI, and rainfall, locational data such as proximity to roads and rivers, socio-economic data including population density, nighttime lights, and various commercial and service aggregation points, as well as meteorological data like annual average temperature and humidity (Supplementary Fig. S1). These features form a feature vector as input to the network. The network initially elevates the dimension of features to 100 through a fully connected layer. Subsequently, it undergoes two self-attention residual modules to perform nonlinear computations, obtaining a feature vector with higher semantic information. The vector's dimension is then reduced to 50 through a series of fully connected layers and another self-attention residual module, densifying the features. The resulting feature vector, with a dimension of 50, is fed through a fully connected layer to calculate scores for three land-use categories. Finally, a softmax function transforms these scores into predicted probabilities for ecological, agricultural, and urban land-use categories. The probabilities of the three categories sum up to 1.

### Experimental design

**Figure 3:**
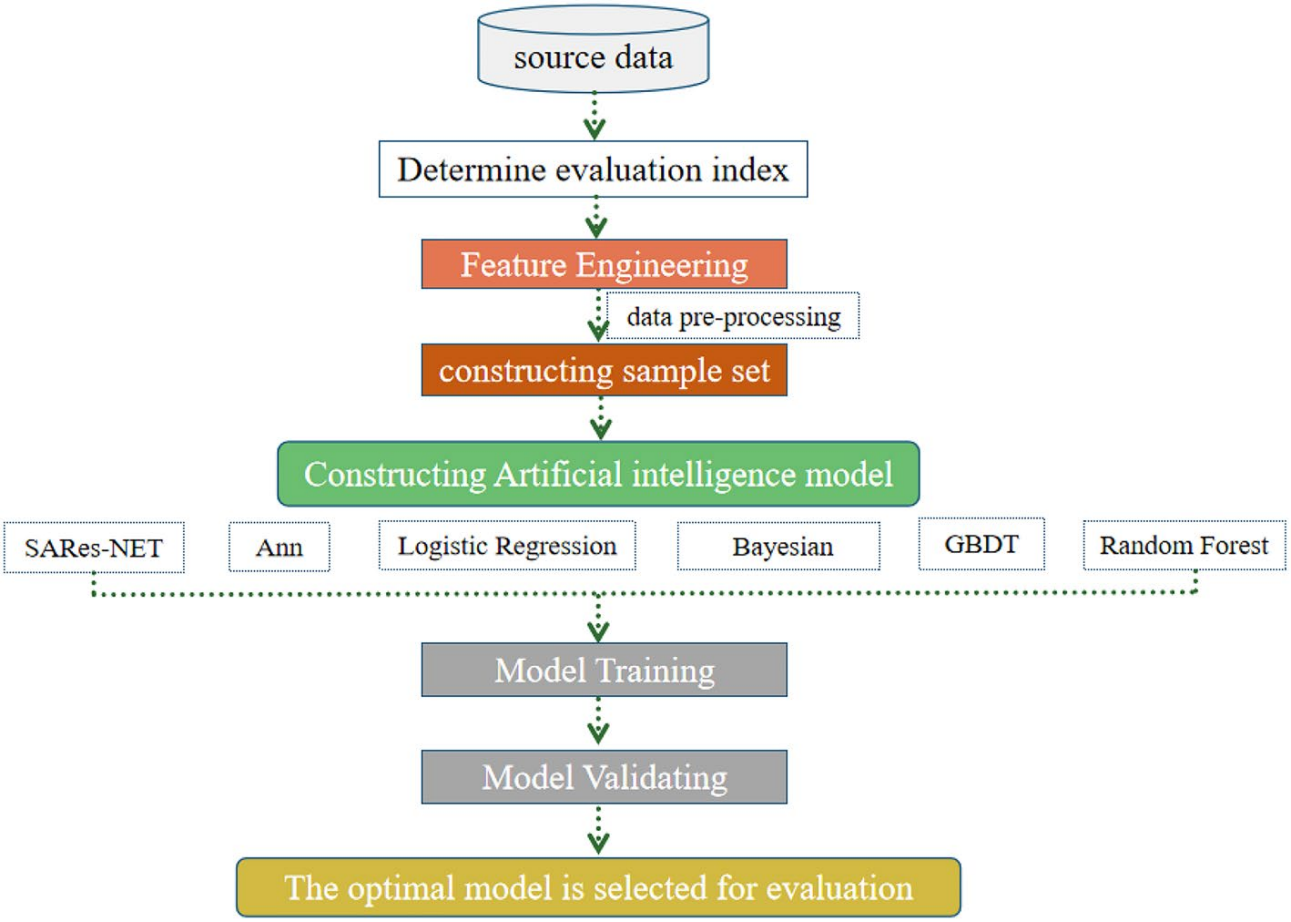
Proposed workflow of our work: We compared SARes-NET with five other models to select the optimal one for spatial evaluation of the three spaces.

This study integrates diverse geographical spatial data for the evaluation of ecological, agricultural, and urban space suitability (see Supplementary Fig. S1 online). Initially, evaluation indicators were selected, and subsequent data preprocessing facilitated the construction of a comprehensive sample set. To assess the E-A-U space, a comparative validation was performed using an array of models, including ANN, GBDT, LR, SARes-NET, NB, and RF algorithms. This comparison aimed to identify the most optimal model. The selected superior model, as determined through the validation process, was then employed for the in-depth evaluation of the E-A-U space, as illustrated in Fig. 3.

#### Dataset construction

(1) Aggregation of multisource spatial data: Uniformly aggregate the data to a spatial resolution of 90 m × 90 m grids, constructing a feature matrix. The entire study area is subdivided into 5,298,221 units. (2) E-A-U Spatial Sample Division: The ecological protection red line, nature reserves, and drinking water sources are vital ecological areas designated by the Chinese government. In this study, these areas serve as ecological space samples (1,052,712). Agricultural space samples (983,306) are selected within permanent basic farmland, while urban space samples (60,961) focus on the existing built-up areas of cities, acknowledging their limited potential for change. (3) Division of Dataset Based on Stratified Sampling: To ensure consistency between the test and training samples, a 7:3 stratified sampling is employed to divide the samples into training and testing sets. The training set is used for model training, while the testing set is employed for model validation and performance evaluation.

#### Model evaluation meric

To assess model performance comprehensively, this study employs a combination of confusion matrix and ROC curve with AUC area to evaluate the classification performance of the model^44^ (see Supplementary Method). Based on the confusion matrix, we calculated the model's accuracy (ACC), precision (PRE), recall (REC), and Kappa coefficient separately for performance comparison^45^. The specific meanings of these indicators are detailed in the Supplementary Method.

#### Implementation details

The model was trained on a 64-bit Windows 10 operating system using the Python programming language, the Pytorch 1.12.0 deep learning framework, and CUDA 11.3. The GPU model used was NVIDIA RTX A4000 (16 GB VRAM), and the CPU model was an 8-core, 16-thread Intel(R) Xeon(R) W-2245 CPU @ 3.90 GHz with a total memory size of 64 GB.

### Mutual information method

Mutual information (MI), rooted in the realm of information theory, was initially introduced by Claude Shannon in his groundbreaking paper, 'A Mathematical Theory of Communication,' in 1948^46^ even though he did not explicitly refer to it as 'mutual information.' The term 'mutual information' was later coined by Robert Fano^47^ MI serves as a measure designed to quantify the level of information exchange between two random variables. Mathematically, MI computes the Kullback–Leibler divergence between the product of the joint probability distribution of two random variables and the marginal probability distribution of these two variables. In practical applications, MI has found extensive use across various domains, particularly in machine learning and data mining. It is frequently employed as a feature selection method to assess feature importance and select the most relevant features by measuring the mutual information between features and target variables^18,48^ The calculation formula is as follows:

1 $${\text{MI}}({{\text{x}}}_{{\text{i}}};{\text{y}})=\sum_{{{\text{x}}}_{{\text{i}}}}\sum_{{\text{y}}}{\text{p}}({{\text{x}}}_{{\text{i}}},{\text{y}}){\text{log}}\frac{{\text{p}}({{\text{x}}}_{{\text{i}}},{\text{y}})}{{\text{p}}({{\text{x}}}_{{\text{i}}}){\text{p}}({\text{y}})}$$

In the formula, '${\text{p}}({{\text{x}}}_{{\text{i}}},{\text{y}})$' represents the joint probability distribution of two variables, '${{\text{x}}}_{{\text{i}}}$' and 'y', while '${\text{p}}({{\text{x}}}_{{\text{i}}})$' and '${\text{p}}({\text{y}})$' represent the marginal probability distribution of '${{\text{x}}}_{{\text{i}}}$' and 'y', respectively. Here, '${{\text{x}}}_{{\text{i}}}$' denotes the i-th input feature, and 'y' represents the label for region division.

## Results

### Modeling validation results

To assess the accuracy of the deep learning model, we conducted validation on the constructed dataset using our network architecture. We compared the predictive performance of our model with five machine learning methods, namely, LR^32^, NB^22^, GBDT^33^, RF^21,41^, and ANN^42^. This evaluation was performed to validate the feasibility of the model in predicting the suitability of the three spatial zones in Yulin City.

**Figure 4:**
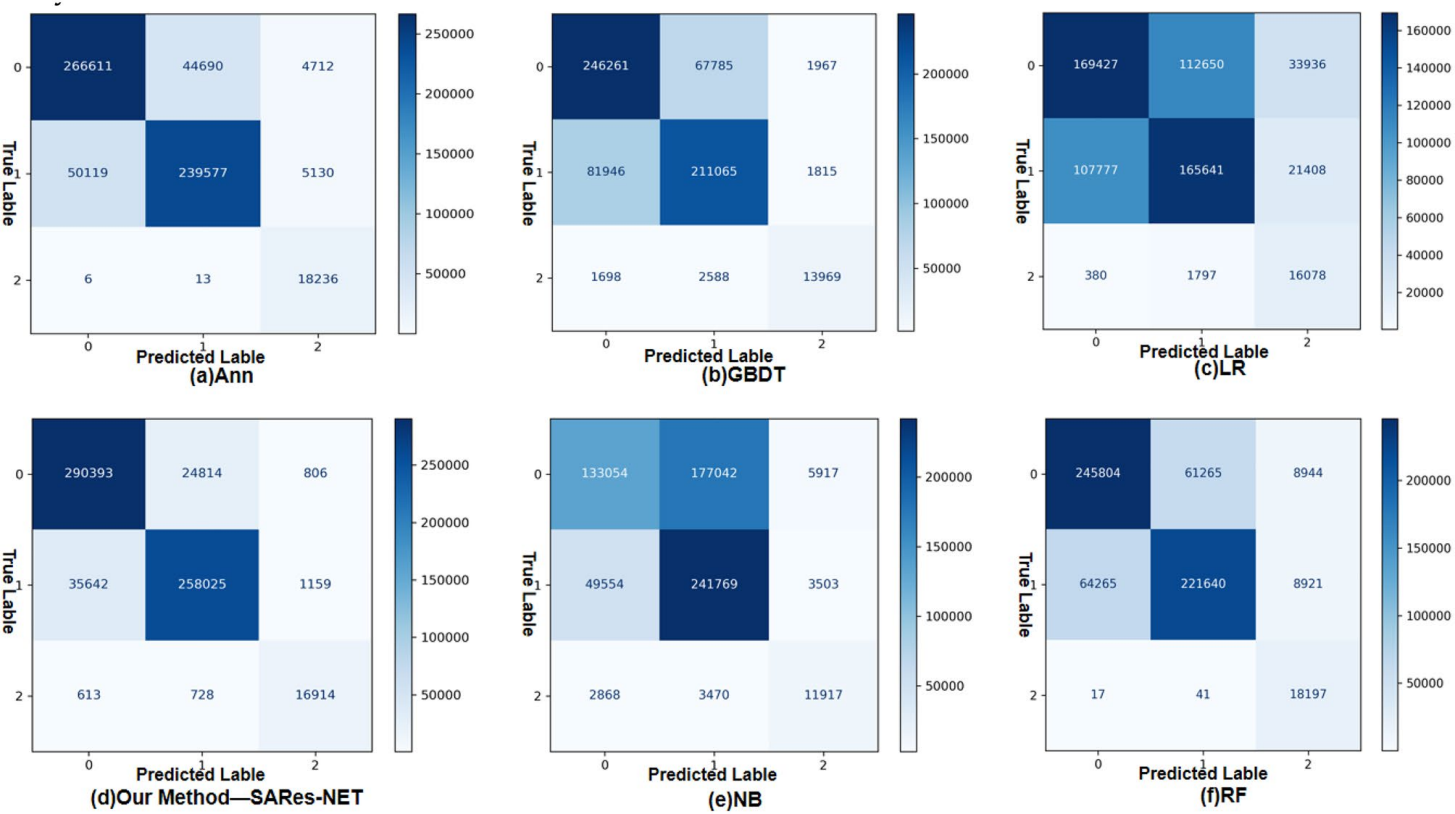
Model Validation Confusion Matrix: (**a**) Artificial Neural Network (ANN); (**b**) Gradient Boosting Decision Trees (GBDT); (**c**) Logistic Regression (LR); (**d**) Self-Attention Residual Neural Network Diagram (SARes-NET); (**e**) Naive Bayes (NB); (**f**) Random Forest (RF). Note that we use ‘0’ as the label for Ecology; ‘1’ as the Agriculture label; ‘2’ as the label for Urban. Each row of the matrix corresponds to a predicted lable, including ecological, agricultural, and urban spaces. Each column of the matrix corresponds to an true lable. The values on the diagonal represent the number of consistent predictions with the ground truth for each category. Compared to other models, our model has the highest number of correct predictions, indicating superior performance of the matrix.

The well-trained model was employed to predict on the validation set, yielding a confusion matrix (Fig. 4). Subsequently, validation set accuracy, Kappa coefficient, precision, and recall were calculated based on Eqs. (1) to (4) outlined in Supplementary Method. As depicted in Table 3, both Ann and our proposed method (SARes-NET) exhibited favorable validation results compared to the other four machine learning models, indicating superior performance of the deep learning model over traditional machine learning models. Notably, the Self-Attention Residual Neural Network designed and implemented in this study outperformed Ann, achieving an accuracy of 89.86%, a Kappa coefficient of 80.77%, precision rates for the ecological, agricultural, and urban models at 88.90%, 90.99%, and 89.59%, respectively, and recall rates of 91.89%, 87.52%, and 92.65%. These results underscore the high simulation accuracy of the model, affirming its applicability within the context of this study.

**Table 3:** Performance comparison table of using six different machine learning and deep learning algorithms (SARes-NET, ANN, RF, GBDT, LR, NB), including Kappa coefficient, accuracy (ACC), recall (REC) and precision (PRE).

	OurMethod-SARes-NET	Ann	RF	GBDT	LR	NB
Kappa	80.77%	68.84%	57.77%	52.32%	21.99%	28.17%
ACC	89.86%	83.36%	77.20%	74.92%	55.82%	61.45%
REC						
Ecological model	91.89%	84.37%	77.78%	77.93%	53.61%	42.10%
Agriculture model	87.52%	81.26%	75.18%	71.59%	56.18%	82.00%
Urban model	92.65%	99.90%	99.68%	76.52%	88.07%	65.28%
PRE						
Ecological model	88.90%	84.17%	79.27%	74.65%	61.04%	71.74%
Agriculture model	90.99%	84.28%	78.33%	74.99%	59.14%	57.25%
Urban model	89.59%	64.95%	50.46%	78.69%	22.51%	55.85%

**Figure 5:**
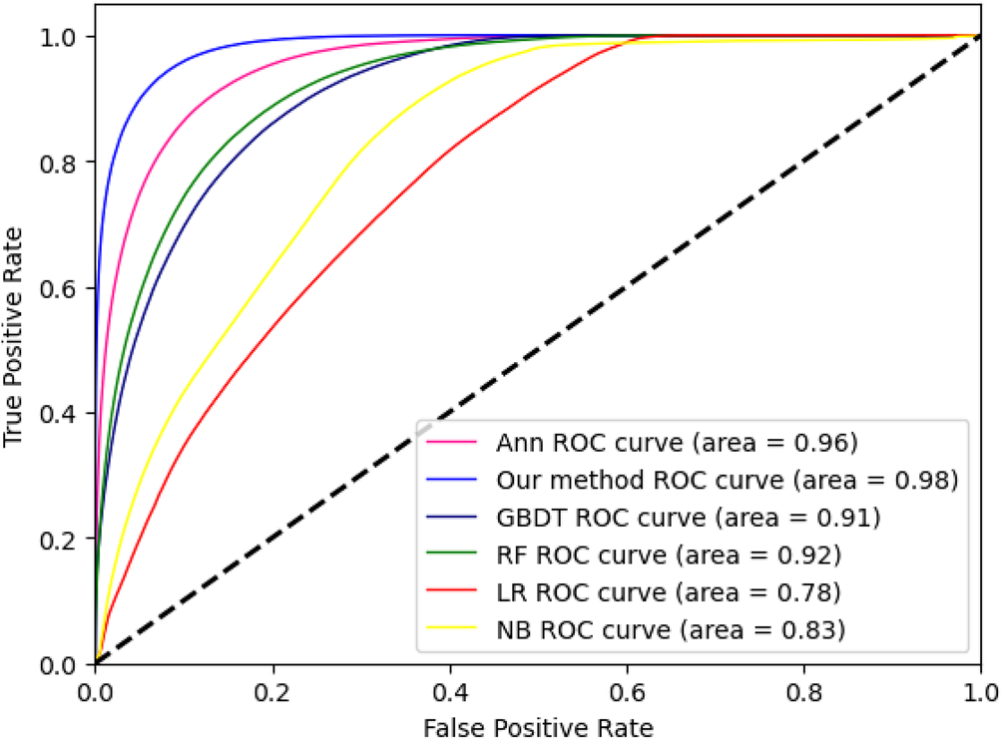
ROC curve Graph of various algorithm models for 'E-U-A' space (ANN, Our method: SARes-NET, GBDT, RF, LR ,NB). The ROC curve closer to the upper left corner indicates a larger area under the curve and better performance of the model.

To comprehensively assess the model's performance, this study employs the ROC curve and the AUC (Area Under the Curve) metric. The model's fitting ROC curve is depicted in Fig. 5, and its corresponding AUC area is calculated. The evident concave trend towards the upper-left of the ROC curve indicates superior classification performance, with a considerable distance from the ROC curve of a purely random classifier (depicted by the black dashed line in the figure). In comparison to other machine learning models, the SARes-NET deep learning model constructed in this study achieves an AUC value of 0.98, surpassing other models. This result signifies excellent classification performance, reaching an ideal state.

### Spatial evaluation results

#### Spatial distribution characteristics of ecology-agriculture-urban

In this study, the output results of the three spatial categories are classified into three levels using a natural break method. Specifically, the evaluation of ecological space is categorized as extremely important, important, and generally important. The evaluation of agricultural space is divided into suitable, moderately suitable, and unsuitable, while the evaluation of urban space is classified as suitable, moderately suitable, and unsuitable. The findings reveal that in terms of spatial distribution, the urban-agricultural-ecological space in Yulin City exhibits the following distribution characteristics.

**Figure 6:**
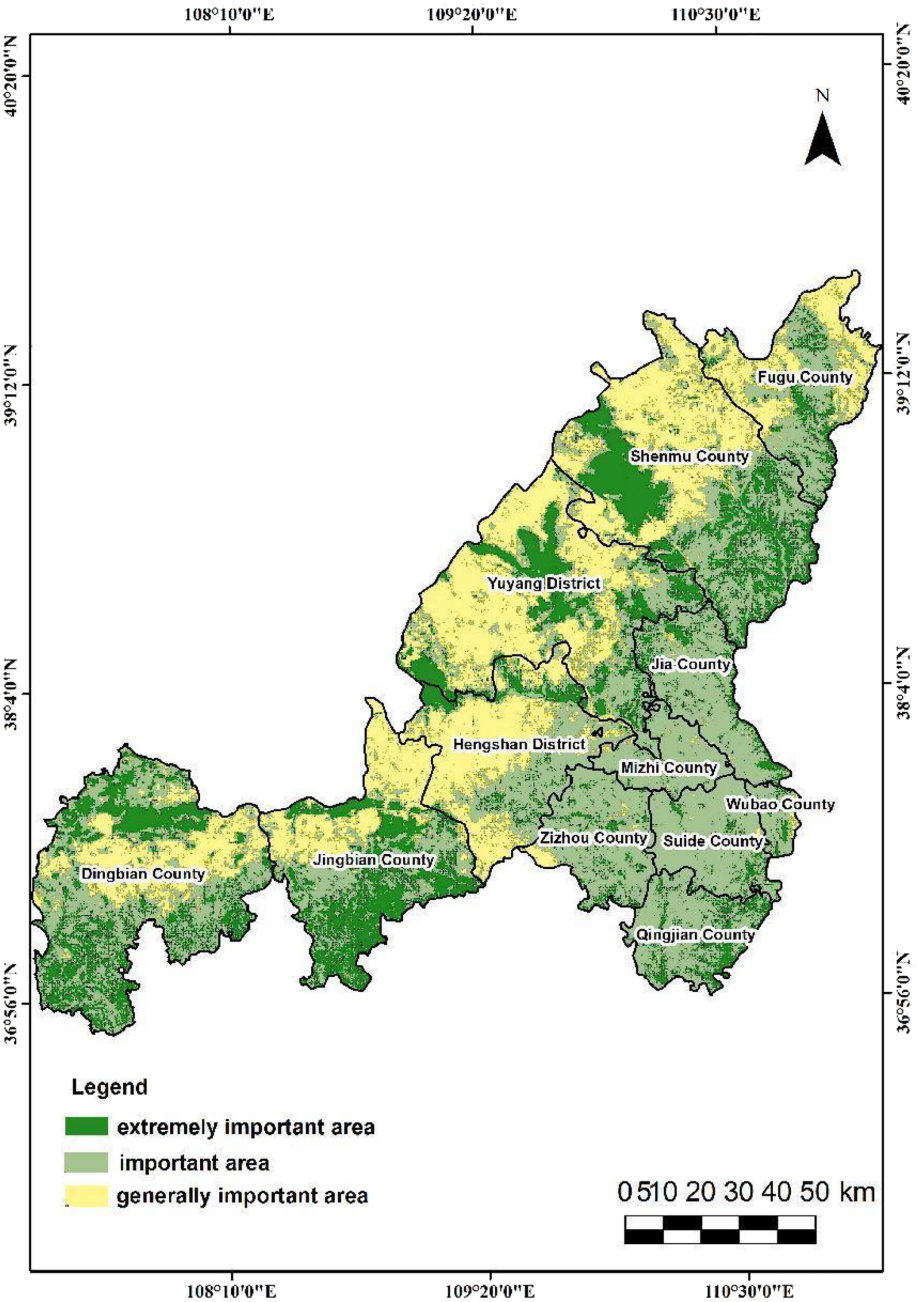
The City of Yulin with the mapped Distribution of Ecological Importance Level of three categories: extremely important area, important area and generally important area, which are drawn by QGIS software (https://qgis.org/en/site/, version: 3.34).

**Figure 7:**
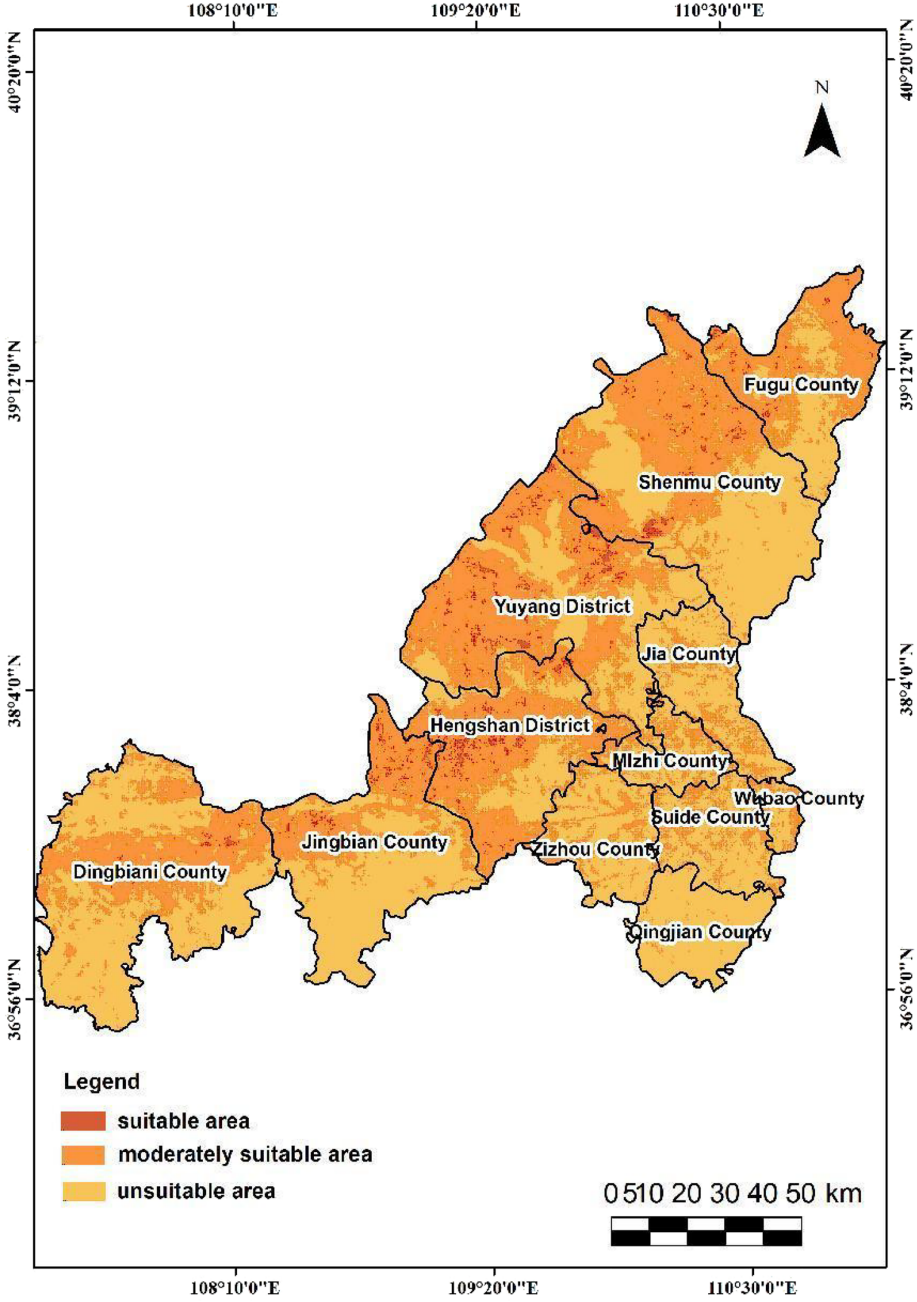
The City of Yulin with the mapped Distribution of Agricultural suitability grade of three categories: suitable area, moderately suitable area and unsuitable area, which are drawn by QGIS software (https://qgis.org/en/site/, version: 3.34).

**Figure 8:**
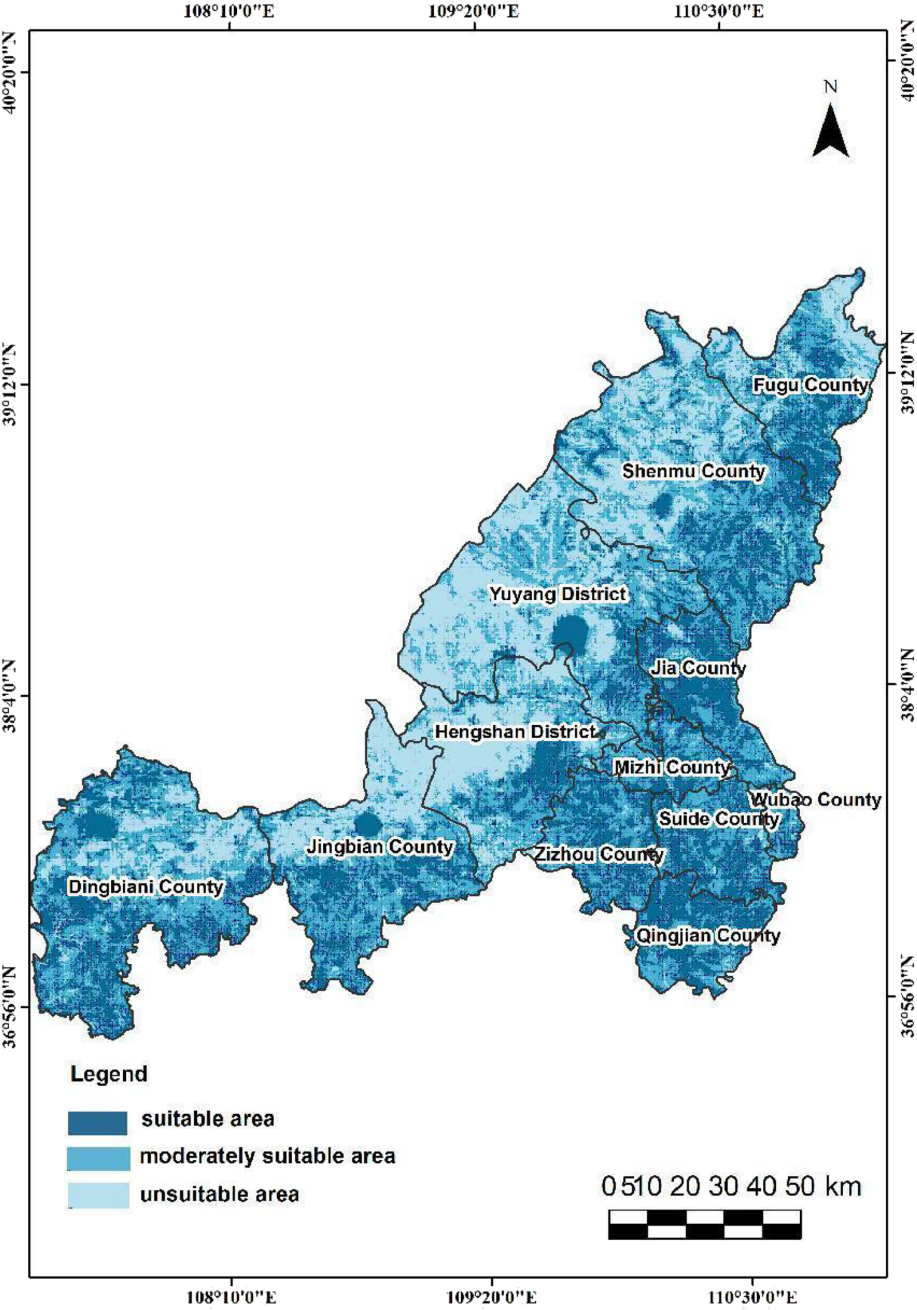
The City of Yulin with the mapped Distribution of Urban suitability grade of three categories: suitable area, moderately suitable area and unsuitable area, which are drawn by QGIS software (https://qgis.org/en/site/, version: 3.34).

1. The extremely important ecological protection area spans 8,978.90 square kilometers, representing 20.92% of the city's total land area. The category with the highest proportion is the important ecological protection area, which accounts for 50.39% of the three categories (Fig. 6). In Yulin City, the moderately suitable area and unsuitable area for agricultural space are relatively similar, covering 40.51% and 58.11% of the territory, respectively(Fig. 7). In contrast, the suitable agricultural area is quite small, comprising only 592.30 square kilometers, or 1.38% of the total region. The second level of urban space(Fig. 8), characterized as the moderately suitable area, is the most extensive, constituting 40.73% of the urban space. The proportion of suitable and unsuitable areas for urban space is approximately equivalent, both standing at about 29%.
2. The northwestern part of the study area is predominantly suitable for agricultural space, with ecological and urban unsuitable areas, and scattered regions of suitable agricultural space. This area exhibits a more evenly distributed average annual rainfall and is closer to water bodies. Land types here are primarily unused and cultivated land, characterized by relatively flat terrain with lower overall slopes compared to the southeast. Soil in this region has low silt content, good texture, and favorable conditions for agriculture, facilitating agricultural development.
3. The eastern segment of the study area can be categorized as ecologically important areas. This region exhibits high vegetation coverage and is primarily comprised of woodland and grassland. Unlike agricultural space, this area does not have extremely important ecological protection areas. Instead, the extremely important ecological protection areas are mainly located in the loess hilly and gully areas of Yulin City, Dingbian County, the southern part of Jingbian County, and the eastern portion of Shenmu. These areas encompass important nature reserves, wetlands, water sources, and other protected regions in the study area. Consequently, this area is classified as unsuitable for both urban and agricultural space.
4. The southeastern part of the study area can be categorized as general urban suitable areas due to its relatively high annual accumulated temperature and humidity, environmental comfort, and suitability for human habitation. The spatial extent of these urban suitable areas aligns closely with the distribution of Point of Interest (POI) data, reflecting concentrations of medical facilities, tourist attractions, educational institutions, and dining establishments. Consequently, this type of area is classified as unsuitable for agriculture, primarily because of its low rainfall and steep slopes, conditions that are not conducive to agricultural cultivation.

#### National territorial spatial pattern distribution in Yulin City

**Figure 9:**
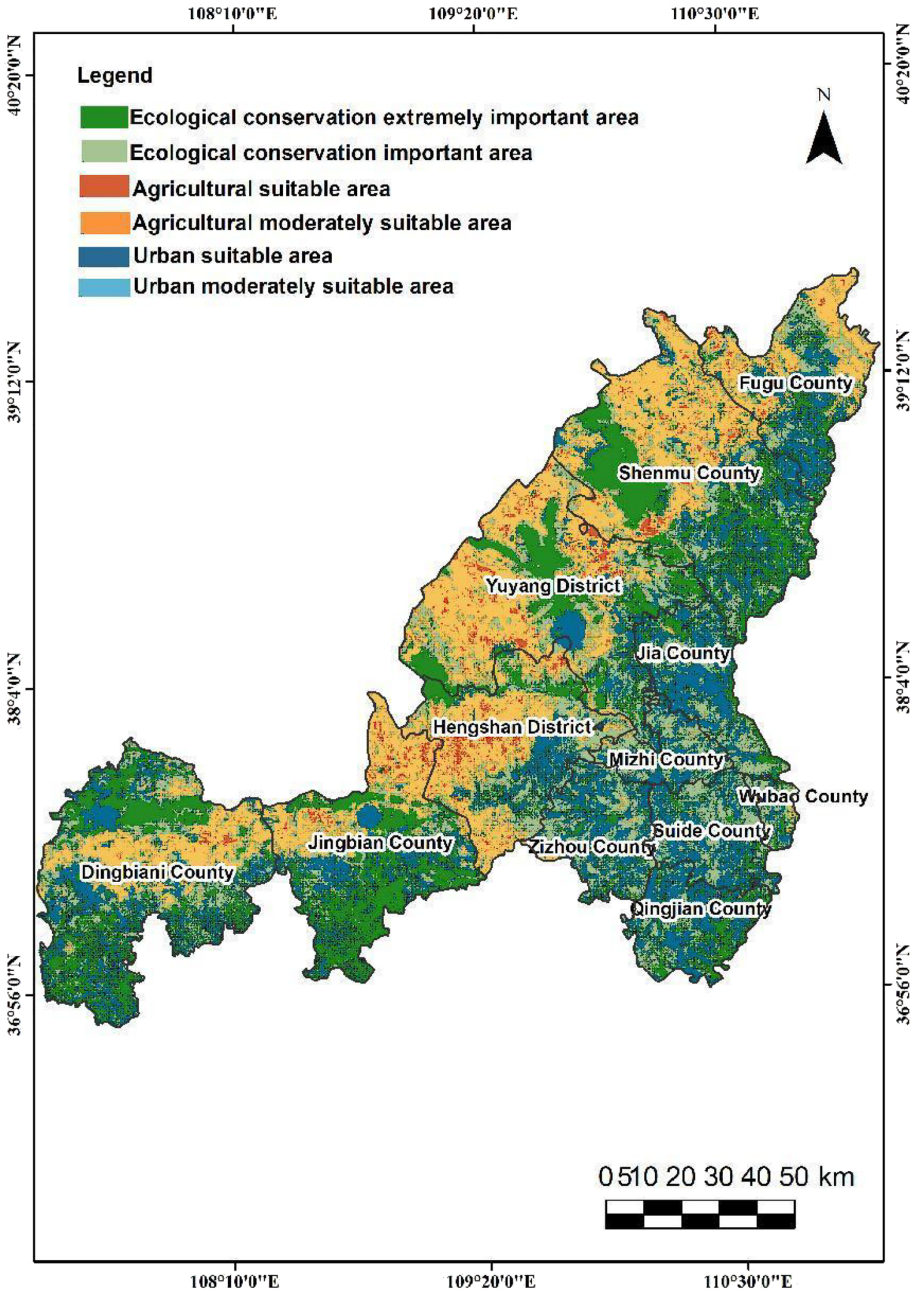
The City of Yulin with the mapped Distribution of National territorial Spatial Pattern of six grade: Ecological conservation extremely important area, Ecological conservation important area, Agricultural suitable area, Agricultural moderately suitable area, Urban suitable area and Urban moderately suitable area, which are drawn by QGIS software (https://qgis.org/en/site/, version: 3.34).

Utilizing the natural breakpoint method, this study classifies the three zones into three distinct levels. Consequently, in the land spatial pattern division, the optimal evaluation outcomes for the three-zone space are preserved, following the principle of 'ecological space > agricultural space > urban space.' The study classifies the region into six categories, each of which is detailed in Table 4. Notably, the extremely important ecological protection areas are primarily located in the northwestern, southwestern, and central regions of Yulin City, encompassing vital nature reserves, wetlands, water sources, and other protected regions within the study area. Ecologically important areas and urban suitable areas are intertwined in the eastern portion of Yulin City. Furthermore, urban suitable areas are predominantly situated in the vicinity of Yuyang and Jingbian. The moderately suitable area for urban space closely resembles the spatial distribution of agricultural space, with a significant presence in the northwestern area of Yulin City. However, there are notable disparities in area distribution, with the agricultural suitability area representing the smallest proportion (Fig. 9).

**Table 4:** National territorial Spatial Pattern distribution map of Yulin City, including the area and proportion of six spatial categories.

Suitability zoning grade	Zoning grade area (km^2^)	Proportion (%)
Ecological conservation extremely important area	898,319.79	20.93
Ecological conservation important area	1,053,261.71	24.54
Agricultural suitable area	592,298.76	13.80
Agricultural moderately suitable area	840,806.72	19.59
Urban suitable area	1,170,433.85	27.27
Urban moderately suitable area	270,397.26	6.30

### Assessment of the significance of driving factors for E-A-U space

**Figure 10:**
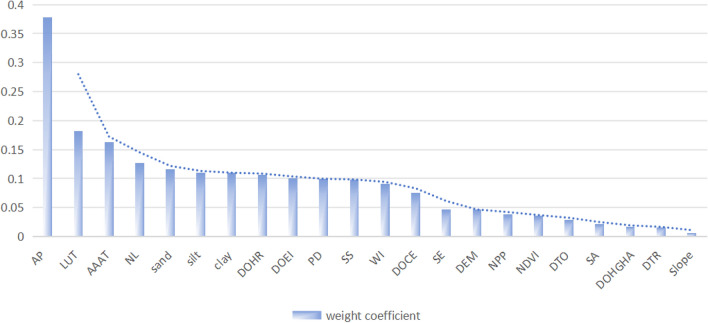
Ranking Chart of driving factor importance of E-U-A Space. Note that AP denotes Average precipitation, LUT denotes Land use types, AAAT denotes Average annual accumulated temperature, NL denotes Night lighting, DOHR denotes Distribution of healthcare regions, DOEI denotes Distribution of educational institutions, PD denotes Population density, SS denotes Scenic spots, WI denotes wetness index, DOCE denotes Distribution of catering establishment, SE denotes Soil erosion, DTO denotes Distance to roads, SA denotes Slope aspect, DOHGHA denotes Distribution of high-risk Geological hazards area, DTR denotes Distance to river.

Mutual information is employed to assess the relationship between multi-class labels and input information. As depicted in Fig. 10, among the 22 influencing factors, rainfall emerges as the most pivotal factor impacting the territorial spatial planning of Yulin City, significantly outweighing other driving factors. Supplementary Figure S1h illustrates the rainfall distribution in Yulin City, demonstrating a gradual decrease from west to east. The eastern part of Yulin City generally experiences moderately weak rainfall, but this area also corresponds to the distribution of urban suitable regions. Insufficient water resources, high population density, and elevated water consumption serve as primary hindrances in this area, rendering ecological and agricultural spaces unsuitable. Furthermore, land use type emerges as a key driving factor shaping the spatial distribution within the study area. Supplementary Figure S1s shows the alignment of forested land and water areas in Yulin City with important ecological protection areas, while cultivated land is concentrated mainly in the Jingbian and Dingbian regions. These areas feature flat terrain, favorable soil texture, and superior agricultural conditions, which is why the evaluation results indicate their significance as agricultural areas. Additionally, the weight of average annual accumulated temperature is relatively high, as depicted in Supplementary Fig. S1(v). The eastern part of Yulin City experiences relatively high average annual accumulated temperatures and fares well in the assessment of ecological and urban spaces, underscoring the importance of climate comfort for human life. Soil texture also exerts a considerable influence on land spatial distribution in Yulin City. Sand, clay, and silty sand are ordered in decreasing significance. Supplementary Figure S1d–f reveals that the northwest of Yulin City has a high sand content and low clay and silty sand content, while the southeast exhibits moderate sand and clay content with high silt content. Consequently, the northwest is predominantly agricultural space, whereas the southeast is primarily urban space. Socio-economic factors, including nighttime lighting, health care facilities, educational institutions, and restaurants, also have a relatively notable impact on the model. Supplementary Figure S1n–r highlights that areas with the highest nighttime lighting are concentrated mainly in Yuyang District, Dingbian, Jingbian, Shenmu, Mizhi, and Suide. Urban space suitable areas are primarily distributed in these regions as well.

## Discussion

The spatial distribution and influencing factors of E-A-U space in Yulin City were analyzed utilizing a multi-source data representation and a deep learning method with GIS technology. The results reveal distinct regional characteristics in the distribution of urban-agricultural-ecological space. In the northwest, agricultural space predominates, suggesting potential adjustments to the agricultural structure based on economic development and regional characteristics. Conversely, in the southeast, urban space and ecological space take precedence, warranting consideration for the establishment of ecological agricultural zones. Extremely important ecological protection areas are primarily situated in Dingbian County, the southern part of Jingbian County, and the eastern region of Shenmu, encompassing essential nature reserves, wetlands, water sources, and other natural protected areas within the study area. The delineation outcomes closely align with the current developmental status of Yulin City and are generally consistent with the functional zoning results at the city and county levels proposed by Gong^49^.

In this study, it was observed that the alterations in E-A-U space are primarily influenced by land resources, natural resources, and social resources, with rainfall, annual accumulated temperature, land use type, soil texture, nighttime lighting, and other factors playing significant roles. Considering the specific conditions of the study area, it can be generally concluded that water resources are the most critical factor affecting the distribution of land space in the region. Yulin City exhibits local vulnerabilities and imbalances in the coordinated development of water resources and regions. Therefore, it is advisable to further adjust the water structure and optimize the allocation of water resources to address these disparities.

This study employs deep learning as an approach for suitability assessment, and the constructed deep learning model achieves an accuracy of 89.86% and a Kappa coefficient of 80.77% on the validation set. In comparison to traditional expert ratings or other subjective weighting methods, this enhances the objectivity of the evaluation results, reinforcing the scientific and practical aspects of suitability assessment. Furthermore, a comparative validation is conducted with five other deep learning and machine learning models, including Ann and GBDT^21,32,33,41,50^. The results of the validation indicate that the performance of the deep learning model surpasses that of machine learning models. Particularly, the SARes-NET model designed and implemented in this study exhibits superior simulation performance compared to Ann, demonstrating its excellent applicability in this context. The model adeptly captures complex nonlinear relationships between evaluation factors and suitability levels, reducing human errors in data processing. This contributes to strengthening the practical application of the outcomes of "Land Suitability Assessment" in guiding the scientific allocation of land space elements and exploring suitability assessment methods tailored for urban scales.

Nonetheless, there are limitations in this study, primarily related to data collection. The acquisition of relevant indicators for land spatial suitability evaluation is not exhaustive, particularly the absence of certain data such as soil heavy metal information and atmospheric data. Furthermore, the study faces data limitations when it comes to water resources data, such as groundwater data and total water consumption. Additionally, the study is confined to a spatial analysis based on 2020 data, and lacks an examination of the temporal dimension. In the future, we aim to address these issues and enhance the comprehensiveness and depth of our research by expanding data sources and considering both spatial and temporal dimensions.

## Conclusions

In the context of ecological civilization construction, land space planning necessitates the scientific, rational, intensive, and efficient utilization of land resources. Land suitability evaluation should adhere to ecological security, resource efficiency, and sustainability standards. In land spatial planning, suitability evaluation serves as a tool for analyzing the foundational conditions of land space. A scientifically sound suitability evaluation can offer robust support for guiding land space planning.

This study drew inspiration from the big data research paradigm and harnessed multi-source data reflecting socio-economic aspects, including nighttime lights and points of interest (POI), to expand the land space suitability evaluation index system. The Self-Attention Residual Neural Network Diagram (SARes-NET) method was compared with five artificial intelligence methods to validate its accuracy and applicability. This approach effectively resolves issues related to multicollinearity among evaluation factors, offering a novel concept for optimizing the spatial pattern of land at the city and county levels. It also provides fresh insights into clarifying zoning evaluation factors and scientific zoning methods.

The northwestern region of the study area is predominantly characterized by agricultural space, offering opportunities for adjustments in the agricultural structure in alignment with economic development and regional features. In the southeast, urban space and ecological space predominate, suggesting potential for the development of ecological agricultural areas. These research findings enhance our comprehension of territorial spatial planning and offer valuable insights for the sustainable development and ecological preservation of Yulin City.

The outcomes of this research offer a fresh perspective on coordinating urban development, ensuring food security, safeguarding ecological integrity, and achieving sustainability. They hold significance as a reference for land space planning and management in other regions and contribute to the advancement of territorial space planning practices, ultimately promoting sustainable development.

### Supplementary Information

Supplementary Information.

## Data Availability

Data are available upon reasonable request, please contact the corresponding author.
