# Supplementary material for: Evaluation method for ecology-agriculture-urban spaces based on deep learning
Source: Sci Rep. 2024 May 18;14:11353. doi: 10.1038/s41598-024-61919-1 (PMC11102479; doi:10.1038/s41598-024-61919-1)
Supplement: Supplementary file 1 — Supplementary Information. [file 41598_2024_61919_MOESM1_ESM.pdf]

Supplementary materials

Evaluation Method for Ecology-Agriculture-Urban Spaces Based on Deep Learning

Anqi Li[1] , Zhenkai Zhang[2,*], Zenglin Hong[134,*],Lingyi Liu[5] and Yuanmin Liu[6]

[1] School of Land Engineering, Chang'an University, Xi an 710054, China.
[2] Shaanxi Satellite Application Center for Natural Resources, Shaanxi Institute of Geological Survey, Xi'an, China.
[3] Satellite Remote Scensing Application Centre, CESS ,China-SCO, Xi'an 710054, China.
[4] Shaanxi Urban Geology and Underground Space Engineering Technology Research Center, Xi'an 710054, China.
[5] School of Computer Science, National Engineering Laboratory for Integrated Aero-Space-Ground-Ocean Big Data Application Technology, Shaanxi Provincial Key Laboratory of Speech Image Information Processing, Northwestern Polytechnical University, Xi'an, 710129, China.
[6] The 41st Institute of the Fourth Academy of CASC , Xi'an 710025, China.

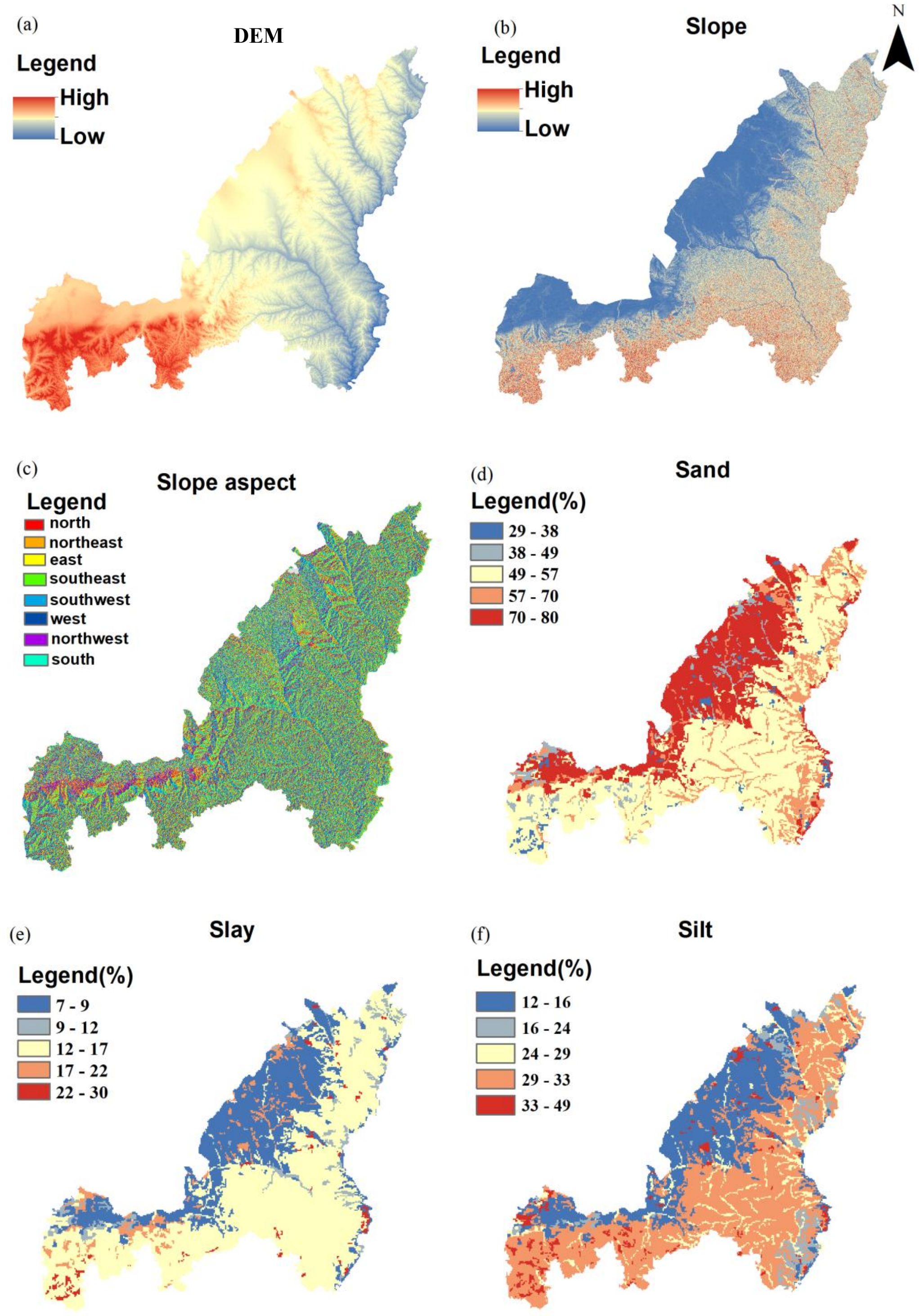
(a)
DEM
Legend
High
Low
(b)
Slope
N
Legend
High
Low
(c)
Slope aspect
Legend
north
northeast
east
southeast
southwest
west
northwest
south
(d)
Sand
Legend(%)
29 - 38
38 - 49
49 - 57
57 - 70
70 - 80
(e)
Slay
Legend(%)
7 - 9
9 - 12
12 - 17
17 - 22
22 - 30
(f)
Silt
Legend(%)
12 - 16
16 - 24
24 - 29
29 - 33
33 - 49

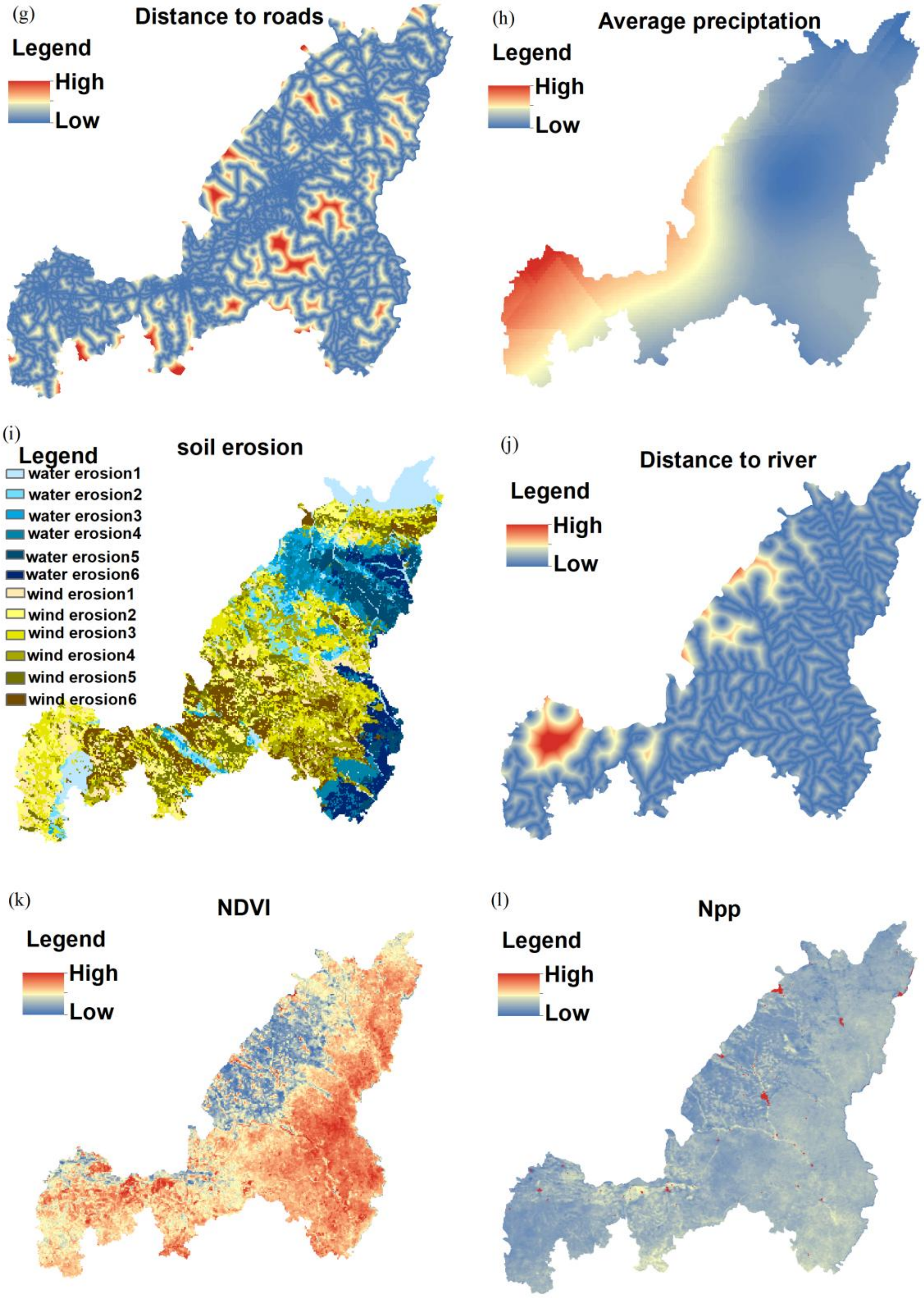
(g)
Distance to roads
Legend
High
Low
(h)
Average preciptation
Legend
High
Low
(i)
soil erosion
Legend
water erosion1
water erosion2
water erosion3
water erosion4
water erosion5
water erosion6
wind erosion1
wind erosion2
wind erosion3
wind erosion4
wind erosion5
wind erosion6
(j)
Distance to river
Legend
High
Low
(k)
NDVI
Legend
High
Low
(l)
Npp
Legend
High
Low

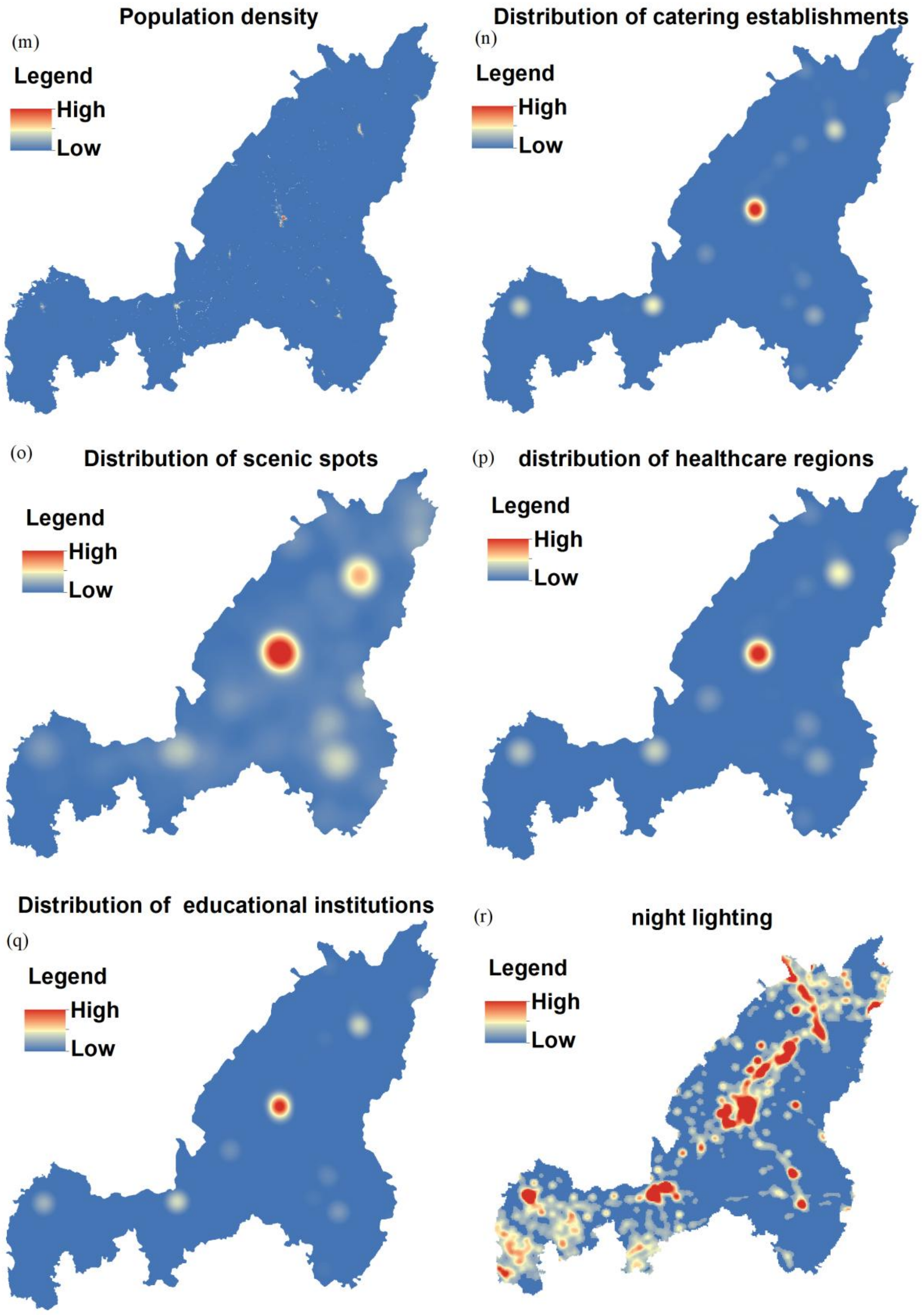
Population density
(m)
Legend
High
Low
Distribution of catering establishments
(n)
Legend
High
Low
(o)
Distribution of scenic spots
Legend
High
Low
(p)
distribution of healthcare regions
Legend
High
Low
Distribution of educational institutions
(q)
Legend
High
Low
(r)
night lighting
Legend
High
Low

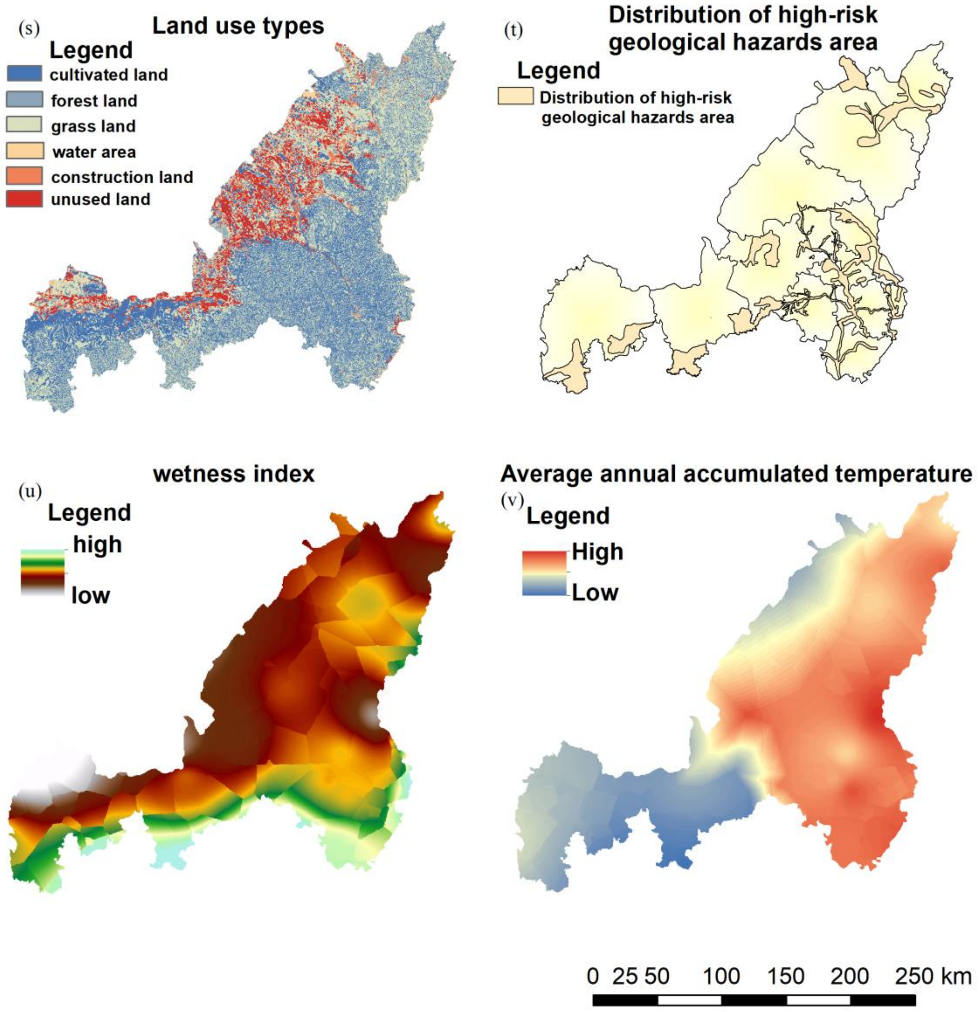

Supplementary Figure S1. The experimental data samples in the study area. Note that we use QGIS sofware (https://qgis.org/en/site/, version: 3.34) for plotting. (a) Dem, (b) Slope, (c) Slope aspect, (d) Sand, (e) Slay, (f) Silt, (g) Distance to roads, (h) Average preciptation, (i) Soil erosion, (j) Distance to river, (k) NDVI, (l) Npp, (m) Population density, (n) Distribution of catering establishments, (o) Distribution of scenic spots, (p) Distribution of healthcare regions, (q) Distribution of educational institutions, (r) Night lighting, (s) Land use types, (t) Distribution of high-risk geological hazards area, (u) wetness index, (v) Average annual accumulated temperature.

Supplementary Method

2.5.2 Model Evaluation Indicators

The confusion matrix, often referred to as the error matrix, is a tabular representation of classification categories. Rows in the matrix represent the classification categories, while columns represent the reference categories (see Supplementary Table S1 online). In the context of simple random sampling, the classification accuracy of the sample is determined by computing the proportion of sample units correctly classified in comparison to both the actual classification and the reference classification. The confusion matrix is instrumental in computing key overall classification accuracy evaluation metrics, including Accuracy (ACC), Precision (PRE), Recall (REC), and the Kappa coefficient.

predicts class						
		Calss 1	Calss 2	...	Calss N	
actual class	Calss 1	a_{11}	a_{12}	...	a_{1N}	$\sum_{k=1}^{N} a_{1k}$
	Calss 2	a_{21}	a_{22}	...	a_{2N}	$\sum_{k=1}^{N} a_{2k}$
	...	...	...	...	...	...
	Calss N	a_{N1}	a_{N2}	...	a_{NN}	$\sum_{k=1}^{N} a_{Nk}$
		$\sum_{k=1}^{N} a_{k1}$	$\sum_{k=1}^{N} a_{k2}$	...	$\sum_{k=1}^{N} a_{kN}$	$\sum_{i,k=1}^{N} a_{ik}$

Supplementary Table S1. Examples of confusion matrix.

(1) Accuracy, ACC

Overall accuracy pertains to the ratio of the total number of correct classifications for all categories (i.e., the sum of the diagonal elements in the confusion matrix) to the total number of reference sample points. It signifies the overall likelihood that the classification outcomes correctly correspond to the actual categories.

$$\mathrm{ACC} = \sum_{k=1}^{r} a_{kk} / N \qquad (1)$$

In the formula, 'r' signifies the total number of rows or columns in the confusion matrix, which is equivalent to the number of categories, and 'N' represents the total number of samples.

(2) Precision, PRE

User precision represents the likelihood that a category is correctly assigned to the specified category during actual classification. It is calculated as the ratio of the number of correct classifications for a specific category (the value on the diagonal within that category's row) to the total number of samples classified within that category (the sum of the data within that category's row).

$$\mathrm{PRE} = \frac{a_{ii}}{a_{+i}} \qquad (2)$$

In the formula, 'a_{ii}' denotes the value located on the diagonal of category 'i' in the confusion matrix, and 'a_{+i}' represents the sum of the values in the corresponding column of category 'i' within the confusion matrix.

(3) Recall, REC

Producer accuracy signifies the likelihood that a category is correctly classified in the actual classification. It is calculated as the ratio of the number of correct classifications for a specific category (the value on the diagonal within that category) to the total number of reference samples belonging to that category (the sum of the data within that category's column).

$$\mathrm{REC} = \frac{a_{ii}}{a_{i+}} \qquad (3)$$

In the formula, 'a_{ii}' denotes the value located on the diagonal of category 'i' in the confusion matrix, and 'a_{+i}' represents the sum of the values within the corresponding column

of category 'i' in the confusion matrix.

(4) Kappa coefficient

The Kappa coefficient assesses the level of agreement between the actual feature category and the classification result category. Unlike overall accuracy, it considers not only the correctly classified instances on the diagonal but also accounts for the misclassifications and missed classifications off the diagonal. The Kappa coefficient falls within the range of [0, 1]. A higher Kappa coefficient value indicates a better classification result.

$$\text{Kappa} = \left(M\sum_{i=1}^{N} a_{ii} - \sum_{i=1}^{N}(a_{i+}a_{+i})\right) \Big/ \left(N^2 - \sum_{i=1}^{N}(a_{i+}a_{+i})\right) \quad (4)$$

In the formula, 'N' represents the total number of rows or columns in the confusion matrix (equivalent to the number of categories), 'a_{ii}' denotes the value on the diagonal of the i-th row and i-th column of the confusion matrix, 'a_{i+}' represents the sum of all the values in the i-th row, 'a_{+i}' represents the sum of all the values in the i-th column, and 'M' represents the total number of samples.

ROC Curve and AUC Area: The ROC curve, or Receiver Operating Characteristic Curve, serves as a comprehensive metric reflecting sensitivity and specificity for continuous variables. It evaluates the model by comparing the performance of true positive rate (TPR) and false positive rate (FPR) at different thresholds. The ROC curve's horizontal axis represents the FPR, while the vertical axis represents the TPR. The enclosed area under the ROC curve, known as the AUC area (Area Under ROC Curve), ranges from 0 to 1, signifying the model's accuracy. A larger AUC value indicates better model performance[1].

$$\text{FPR} = \frac{\text{FP}}{\text{FP+TN}} \quad (5)$$

$$\text{TPR} = \frac{\text{TP}}{\text{TP+FN}} \quad (6)$$

References:

1 Keykhay-Hosseinpoor, M., Kohsary, A., Hossein-Morshedy, A. & Porwal, A., A machine learning-based approach to exploration targeting of porphyry Cu-Au deposits in the Dehsalm district, eastern Iran. *ORE GEOL REV* **116** 103234 (2020).
